# The Role of Phytochemicals in Managing Neuropathic Pain: How Much Progress Have We Made?

**DOI:** 10.3390/nu16244342

**Published:** 2024-12-16

**Authors:** Aleksandar Sic, Aarish Manzar, Nebojsa Nick Knezevic

**Affiliations:** 1 Department of Anesthesiology, Advocate Illinois Masonic Medical Center, Chicago, IL 60657, USA; aca.smed01@gmail.com (A.S.); aarishsyedmanzar@gmail.com (A.M.); 2 Faculty of Medicine, University of Belgrade, 11000 Belgrade, Serbia; 3 Department of Anesthesiology, University of Illinois, Chicago, IL 60612, USA; 4 Department of Surgery, University of Illinois, Chicago, IL 60612, USA

**Keywords:** neuropathic pain, phytochemicals, multiple sclerosis, Guillain-Barré syndrome, diabetic neuropathy

## Abstract

Neuropathic pain is a complex and debilitating condition resulting from nerve damage, characterized by sensations such as burning, tingling, and shooting pain. It is often associated with conditions such as multiple sclerosis (MS), Guillain-Barré syndrome (GBS), and diabetic polyneuropathy. Conventional pain therapies frequently provide limited relief and are accompanied by significant side effects, emphasizing the need to explore alternative treatment options. Phytochemicals, which are bioactive compounds derived from plants, have gained attention for their potential in neuropathic pain management due to their diverse pharmacological properties, including anti-inflammatory, antioxidant, and neuroprotective effects. This review evaluates the mechanisms by which specific phytochemicals, such as curcumin, resveratrol, and capsaicin, influence neuropathic pain pathways, particularly their role in modulating inflammatory processes, reducing oxidative stress, and interacting with ion channels and signaling pathways. While curcumin and resveratrol are primarily considered dietary supplements, their roles in managing neuropathic pain require further clinical investigation to establish their efficacy and safety. In contrast, capsaicin is an active ingredient derived from chili peppers that has been developed into approved topical treatments widely used for managing neuropathic and musculoskeletal pain. However, not all phytochemicals have demonstrated consistent efficacy in managing neuropathic pain, and their effects can vary depending on the compound and the specific condition. The pathophysiology of neuropathic pain, involving maladaptive changes in the somatosensory nervous system, peripheral and central sensitization, and glial cell activation, is also outlined. Overall, this review emphasizes the need for continued high-quality clinical studies to fully establish the therapeutic potential of phytochemicals in neuropathic pain management.

## 1. Introduction

Neuropathic pain, a complex and often debilitating condition resulting from nerve damage, is characterized by sensations such as burning, tingling, and shooting pain [1]. This condition is challenging to treat with conventional therapies, which often provide limited relief and are associated with side effects that significantly impact patients’ quality of life [2]. Moreover, emotional, and psychological factors, including depression and anxiety, frequently coexist with neuropathic pain, exacerbating pain perception and further diminishing overall well-being [3]. Current pharmacological approaches, including antidepressants, anticonvulsants, and opioids, frequently fail to achieve long-term efficacy in neuropathic pain management [4]. Consequently, there is a growing urgency to explore alternative treatment options that can offer both efficacy and safety.

Phytochemicals, bioactive compounds derived from plants, have garnered significant interest as potential agents for neuropathic pain management [5]. These compounds, which include flavonoids, terpenoids, alkaloids, and phenolic acids, exhibit a range of pharmacological properties, including antioxidant, anti-inflammatory, and neuroprotective effects [6]. Notably, several phytochemicals can modulate pain pathways by influencing mechanisms including oxidative stress, inflammation, and ion channel activity- all of which play a role in neuropathic pain pathophysiology [7].

Recent research suggests that certain phytochemicals hold promising therapeutic potential, potentially by targeting specific molecular mechanisms associated with neuropathic pain [8]. For example, curcumin, resveratrol, and capsaicin have shown potential in preclinical studies, showing effects such as modulation of inflammatory cytokines, reduction in oxidative stress, and interaction with nociceptive receptors, which may contribute to pain relief [9].

This paper aims to examine the mechanisms by which phytochemicals influence neuropathic pain pathways and evaluate their therapeutic potential, addressing an important question: Are we on the verge of discovering new solutions? This will be further discussed in the sections that follow.

## 2. Phytochemicals: Definition and Classification

Phytochemicals are bioactive compounds found in plants that contribute to their color, flavor, and disease resistance [10]. These compounds are not essential for basic plant growth, but they can offer health benefits when consumed by humans. Phytochemicals are broadly classified into two major categories: primary metabolites and secondary metabolites [11]. Primary metabolites, such as carbohydrates, proteins, and lipids, are involved in essential growth processes. Secondary metabolites, on the other hand, are not directly involved in growth but play a crucial role in plant defense mechanisms, helping to protect plants from pests, diseases, and environmental stresses [11].

Secondary metabolites, which include alkaloids, flavonoids, terpenoids, phenolic acids, and glucosinolates, are of particular interest due to their potential therapeutic effects [11]. These compounds are often studied for their antioxidant, anti-inflammatory, and anticancer properties, and many have shown promise in treating or managing chronic diseases, including neuropathic pain. Understanding the classification and properties of phytochemicals is essential for exploring their potential role in pain management and other health benefits [12,13].

## 3. Epidemiology and Economic Burden of Neuropathic Pain

Neuropathic pain affects millions globally, with epidemiological data indicating a prevalence of approximately 7–10% in the general population [14,15]. Higher rates are observed among specific groups, such as those with diabetes, multiple sclerosis (MS), and postherpetic neuralgia [16]. Diabetic neuropathy alone impacts up to 50% of patients with diabetes, making it one of the most common causes of neuropathic pain worldwide [17].

Beyond its high prevalence, neuropathic pain imposes a substantial economic burden, encompassing both direct and indirect costs [18]. One study reported that the average annual cost per patient for neuropathic pain management is approximately $4706, underscoring the need for more effective treatments to ease these financial pressures on patients and health systems [19].

In addition to direct healthcare expenses, indirect costs further compound the economic strain, as reduced productivity, absenteeism, and, in severe cases, long-term disability are common [20]. Many neuropathic pain patients experience functional impairments that limit their ability to work or perform daily activities, leading to diminished quality of life. Psychological comorbidities, including depression and anxiety, are also prevalent and add to the condition’s personal and economic burdens [20,21].

## 4. Mechanisms in Neuropathic Pain

### 4.1. Pathophysiology of Neuropathic Pain

Neuropathic pain arises from damage or dysfunction within the somatosensory nervous system, leading to abnormal pain signaling. Unlike nociceptive pain, which results from tissue injury and engages protective reflexes [22], neuropathic pain persists due to maladaptive changes in both the peripheral and central nervous systems. These include abnormal excitability and spontaneous discharges in injured and uninjured neurons, driven by increased expression of voltage-gated sodium channels, resulting in continuous or intermittent pain sensations, often described as burning, shooting, or stabbing [23].

A key feature of neuropathic pain is peripheral sensitization, where damaged nerves release pro-inflammatory cytokines and chemokines that attract immune cells to the injury site. This inflammation amplifies pain sensitivity by upregulating ion channels and altering receptor functions in primary afferent neurons. Central sensitization involves heightened responsiveness of neurons in the spinal cord and brain, facilitated by excitatory neurotransmitters such as glutamate, which act on NMDA and AMPA receptors, increasing synaptic plasticity and amplifying pain signals [24].

Glial cells, particularly microglia and astrocytes, play a crucial role in neuropathic pain by releasing inflammatory mediators and altering synaptic transmission [25]. Microglia become activated in response to nerve injury, releasing pro-inflammatory cytokines that perpetuate central sensitization and worsen pain. Disruption of inhibitory pathways, such as those involving GABA and glycine, exacerbates pain, as they fail to suppress nociceptive signaling effectively [26].

Neuropathic pain is also associated with altered expression of ion channels and receptors involved in pain perception. Upregulation of TRPV1 receptors in sensory neurons increases sensitivity to temperature and mechanical stimuli, further enhancing pain perception [27]. Prolonged peripheral input leads to long-term changes in gene expression in the central nervous system, contributing to a chronic pain state [27].

### 4.2. Role of Inflammation and Oxidative Stress

Inflammation and oxidative stress are major contributors to the progression and persistence of neuropathic pain. Following nerve injury, pro-inflammatory cytokines (e.g., TNF-α, IL-1β, and IL-6) are released, sensitizing peripheral nociceptors [28]. Inflammatory cells infiltrate the injury site, exacerbating inflammation and promoting reactive oxygen species (ROS) release as part of the immune response [29].

Oxidative stress, characterized by an excess of ROS and reactive nitrogen species (RNS), damages cellular components such as lipids, proteins, and nucleic acids, impairing mitochondrial function and disrupting neuronal energy balance [30]. ROS accumulation in sensory neurons activates pathways that upregulate pro-inflammatory genes, further sensitizing neurons [31].

Chronic activation of microglia and astrocytes amplifies central sensitization by releasing additional pro-inflammatory cytokines, nitric oxide, and ROS, increasing excitatory neurotransmission in the spinal cord [32]. Astrocytes increase glutamate release and reduce its uptake, contributing to heightened pain perception and the maintenance of chronic pain [32].

Oxidative stress also depletes endogenous antioxidants, such as glutathione, exacerbating cellular damage and perpetuating a cycle of neuroinflammation and oxidative stress that sustains neuropathic pain [33].

### 4.3. Nociceptive Pathways and Pain Perception

Pain, particularly in neuropathic conditions, arises from the activation of pathways carrying signals from sensory receptors to the brain. Nociceptive pain begins when nociceptors detect harmful stimuli such as mechanical pressure, temperature extremes, or chemical irritants [34]. These signals are transmitted via afferent nerve fibers to the spinal cord, where they are modulated before being relayed to higher brain centers [34].

Two main nociceptive fiber types are involved: A-delta fibers (myelinated, rapid transmission of sharp pain) and C fibers (unmyelinated, slower transmission of dull, aching pain) [35]. The combination allows for immediate and persistent pain responses, common in neuropathic pain [35].

Upon reaching the spinal cord, nociceptive signals synapse with second-order neurons in the dorsal horn, where neurotransmitters such as substance P, glutamate, and CGRP amplify pain signals, contributing to central sensitization. This heightened sensitivity leads to pain perception disorders such as hyperalgesia and allodynia [34].

Second-order neurons project to the thalamus, from where pain and temperature signals are processed and relayed to cortical areas responsible for sensory discrimination and emotional response [36].

In neuropathic pain, dysfunction within these pathways, often due to sustained peripheral nerve damage and central sensitization, can lead to “rewiring” of nociceptive circuits, making pain persistent and less responsive to conventional analgesics [37].

## 5. Neuropathic Pain in Neurological Conditions

Neuropathic pain is a common symptom across various neurological conditions, including multiple sclerosis (MS), Guillain-Barré syndrome (GBS), and diabetic polyneuropathy [16]. In MS, pain arises from the demyelination of nerve fibers, disrupting normal sensory processing and leading to abnormal pain perception [38]. GBS, which involves peripheral nerve damage and inflammation, can also trigger neuropathic pain, often experienced as tingling, burning, or shooting sensations [1,16]. Diabetic polyneuropathy, a frequent complication of diabetes, is caused by nerve damage due to prolonged hyperglycemia, leading to debilitating pain that significantly impacts quality of life [39].

### 5.1. Guillain-Barré Syndrome (GBS)

Guillain-Barré Syndrome (GBS) is an acute inflammatory polyneuropathy that primarily affects the peripheral nervous system, typically presenting as a monophasic illness [40]. It is characterized by rapid-onset muscle weakness and paralysis, often preceded by an infection [41]. GBS is an immune-mediated disorder in which the body’s immune system mistakenly attacks the myelin sheath or axons of peripheral nerves, leading to widespread inflammation and demyelination. In addition to motor symptoms, pain is a common feature of GBS, frequently manifesting as severe neuropathic pain that can persist even after other neurological symptoms have improved [42].

#### Pathophysiology and Clinical Presentation of Pain in GBS

Pain in GBS is often overlooked in clinical practice, leading to inadequate treatment and a diminished quality of life for patients [43]. It arises from inflammation, demyelination, and axonal degeneration of peripheral nerves [44].

In GBS, the immune system primarily targets the myelin sheath of peripheral nerves, causing demyelination [45]. This disruption leads to erratic and hypersensitive nerve signaling in sensory neurons, resulting in hyperalgesia and allodynia [1]. In severe cases, axonal degeneration disrupts nerve conduction, intensifying pain even in the absence of external stimuli [46]. Prolonged nerve injury can also lead to central sensitization, where nociceptive input from damaged nerves sensitizes spinal cord neurons, amplifying pain [47]. Additionally, changes in sodium channels due to demyelination and inflammation contribute to hyperexcitability and erratic pain signaling [48].

The pain in GBS varies significantly, with many patients describing it as sharp, burning, or stabbing, commonly in the lower extremities but also affecting the back and upper limbs [49]. This pain is often resistant to standard analgesics, complicating management. Myalgia, joint pain, and radicular pain may also be present, disrupting sleep and quality of life. Nerve sensitization can increase sensitivity to touch, heightening discomfort and anxiety [46,50,51].

### 5.2. Multiple Sclerosis (MS)

Multiple Sclerosis (MS) is a chronic autoimmune disorder where the immune system attacks the central nervous system, causing inflammation, demyelination, and axonal degeneration [52]. Neuropathic pain affects 50–75% of MS patients and severely impacts their quality of life [53,54]. According to the MS Atlas 2020, around 2.8 million people worldwide are living with MS [55], underscoring the need for effective management strategies for this population. MS patients, especially those with additional conditions such as seizures or epilepsy, often experience a significant reduction in their quality of life [56].

Neuropathic pain in MS differs from nociceptive pain, originating from nerve damage, and can manifest as dysesthetic pain (burning, tingling), allodynia (pain from non-painful stimuli), and hyperalgesia (increased pain sensitivity) [56]. This chronic pain often worsens fatigue, sleep issues, and depression, complicating treatment approaches [57].

#### Mechanisms of Neuropathic Pain in MS

The mechanisms of neuropathic pain in MS involve both peripheral and central components. At the periphery, demyelination disrupts nerve conduction, causing sensory neuron hyperexcitability and spontaneous pain signals. Immune-mediated inflammation releases pro-inflammatory cytokines, sensitizing peripheral nociceptors. Changes in ion channel expression, especially increased sodium and calcium channel activity, further enhance neuronal excitability and pain signaling [58].

Central sensitization also plays a role, as prolonged nociceptive input from damaged peripheral nerves alters spinal cord and brain pain responses, resulting in hyperactivity of dorsal horn neurons and an exaggerated pain response [37].

### 5.3. Diabetic Polyneuropathy (DP)

Diabetic polyneuropathy affects about 50% of people with diabetes [59], with 25% experiencing pain as the primary symptom [60]. It is caused by prolonged hyperglycemia, which leads to nerve damage through oxidative stress, inflammation, and impaired circulation [60,61]. Symptoms include numbness, tingling, burning sensations, and sharp pain, particularly in the extremities, severely affecting daily activities and emotional well-being [21]. The condition also increases the risk of falls, infections, and other complications, underlining the importance of effective management [62].

#### Impact of Diabetes on Nerve Health

In individuals with diabetes, nerve health is greatly affected by both metabolic and vascular factors. Chronic hyperglycemia leads to the accumulation of advanced glycation end products (AGEs), which damage nerve fibers and impair normal nerve function [63]. Oxidative stress, resulting from elevated glucose levels, further contributes to neuronal injury as ROS accumulate and disrupt nerve tissue and repair processes [64]. Vascular complications, such as reduced blood flow and endothelial dysfunction, worsen nerve damage by limiting nutrient and oxygen supply to the nerves. Over time, this leads to the progressive deterioration of nerve function seen in diabetic polyneuropathy [64].

## 6. Neuropathic Pain Management

Neuropathic pain is often treated with antiepileptic drugs (AEDs) such as gabapentin and pregabalin, which reduce nerve excitability via calcium channel binding. Other AEDs, such as lamotrigine and carbamazepine, block sodium channels, while lacosamide enhances sodium channel inactivation. Valproate and clonazepam affect GABAergic neurotransmission, and topiramate combines sodium channel blockade with enhanced GABA function. However, AEDs can cause cognitive and motor impairments, and serious side effects such as blood disorders may occur [65].

Amitriptyline, an antidepressant, is sometimes used, but its effectiveness is limited. It may provide pain relief in about 25% more patients than placebo, though side effects make it less ideal for long-term use [66]. Tricyclic antidepressants (TCAs), such as nortriptyline, may enhance pain relief when combined with other agents such as morphine [67].

Duloxetine is effective for diabetic peripheral neuropathy, regulating pain pathways by inhibiting serotonin and norepinephrine reuptake. It is well-tolerated, though higher doses can lead to more side effects, with serious adverse effects being rare [68].

For resistant cases, opioids and tramadol are prescribed. Tramadol offers modest pain relief but has common side effects such as dizziness and nausea, and opioids carry risks of tolerance, dependence, and other side effects [69,70].

Topical agents, such as lidocaine patches, and NSAIDs, are options, though NSAIDs are less effective for neuropathic pain [71]. Combination therapies, such as pregabalin with TNF-α blockers, enhance anti-nociceptive effects and treatment tolerability [72].

Non-pharmacological approaches, including physical therapy, occupational therapy, and complementary treatments such as transcutaneous electrical nerve stimulation (TENS), can improve mobility and quality of life [73]. Neuromodulation techniques, such as spinal cord stimulation and transcranial magnetic stimulation (TMS), are being explored for patients who do not respond to traditional treatments [74].

Non-pharmacological approaches, such as physical and occupational therapy, TENS, spinal cord stimulation, and transcranial magnetic stimulation (TMS), may improve the quality of life for some patients [73,74]. Lifestyle changes, including glycemic control and physical activity, are important for preventing further nerve damage [75]. Patient education on self-management and adherence is crucial for long-term care [76].

Recent research also highlights AMPK dysfunction in pain mechanisms, particularly in Guillain-Barré syndrome, with therapies such as metformin showing potential neuroprotective effects [77].

**Figure 1:**
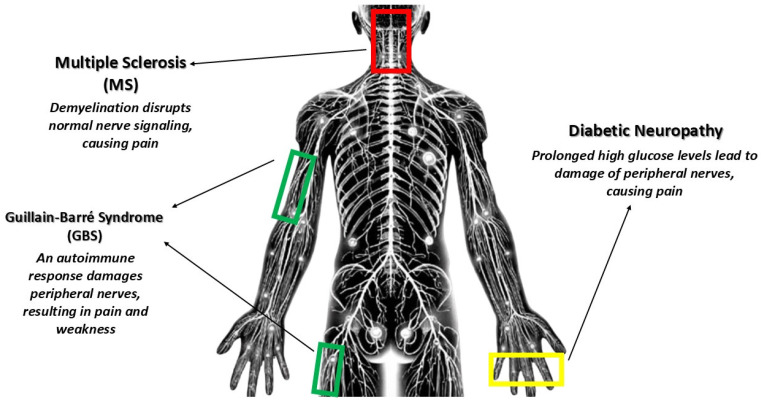
Mechanisms of neuropathic pain in MS, GBS, and diabetic neuropathy.

Figure 1 represents a schematic illustration of the mechanisms of neuropathic pain in diabetic neuropathy, MS, and GBS. It shows distal peripheral nerve damage due to high glucose levels in diabetic neuropathy, demyelination and abnormal signaling in MS, and an autoimmune response affecting peripheral nerves in GBS.

## 7. Phytochemicals in Neuropathic Pain Management

### 7.1. Flavonoides

Flavonoids are a crucial class of secondary metabolites, distinguished by a benzopyrone structure with phenolic or polyphenolic groups. These compounds contribute significantly to plant medicinal properties and biological functions [13,78]. Flavonoids, such as Narirutin, a natural compound derived from *Citrus unshiu*, demonstrate significant therapeutic potential in neuropathic pain management. Specifically, Narirutin exerts its antinociceptive effects by selectively targeting Nav1.7 voltage-gated sodium channels, a mechanism that highlights its promise as a small-molecule treatment option [79].

Flavonoids, such as diosmin, quercetin, and 6-methoxyflavanone, modulate a range of biological pathways that are critical for the management of neuropathic pain. These mechanisms include modulation of inflammatory cytokines, neurotransmitter systems, and oxidative stress responses. Diosmin, a glycoside found in citrus fruits and derived from hesperidin, exhibits antioxidant, antidiabetic, anti-inflammatory, and anticancer properties. Its anti-inflammatory effects are linked to its ability to suppress overexpression of NF-κB, TNF-α, COX-2, and iNOS [80]. Diosmin may also inhibit neuroinflammation by modulating glial cell activity and reducing the production of pro-inflammatory cytokines such as IL-1β. In chronic constriction injury (CCI) models in mice, diosmin has shown potential in treating neuropathic pain through the NO/cGMP/PKG/KATP channel pathway, modulation of glial cells, and inhibition of spinal cytokines such as IL-1β, resulting in analgesic effects via dopaminergic and opioidergic pathways [81].

Quercetin has been shown to exert antinociceptive effects in diabetic neuropathic pain (DNP), likely through modulation of the opioidergic system [82]. Additionally, quercetin reduces microglia and astrocyte activation, mitigating neuroinflammation, which is crucial for preventing the progression of chronic pain. By inhibiting pathways such as TLR4/NF-κB and modulating apoptotic markers, quercetin promotes neuroprotection and reduces neuronal damage, ultimately improving memory and cognitive function in models of neuropathic pain [83].

6-Methoxyflavanone (6-MeOF) has shown potential in attenuating diabetic neuropathic pain and vulvodynia through interaction with GABAergic and opioidergic systems, which likely contributes to its analgesic properties and reduction in allodynia and vulvodynia in animal models [6].

The combination of Berberine and Tocopherol provides a multifaceted approach to managing diabetic neuropathy through a combination of antioxidant effects and modulation of glucose metabolism. Berberine enhances insulin secretion and peripheral glucose utilization, while tocopherol protects neuronal cells from oxidative damage, supporting neuroprotective and anti-inflammatory effects in pain management [84].

### 7.2. Terpenoids

Terpenoids are gaining attention for their potential in neuropathic pain management, thanks to their diverse pharmacological properties. These compounds modulate neurotransmitter systems, receptor activity, and inflammatory pathways, making them promising candidates for chronic pain syndromes [85].

A significant feature of terpenoids is their ability to interact with cannabinoid receptors, particularly CB2, which are crucial for pain modulation. These compounds may act as modulators of pain signaling pathways without inducing the psychoactive effects typically associated with cannabinoid therapies. These compounds may act as modulators of pain signaling pathways without inducing the psychoactive effects typically associated with cannabinoid therapies [86,87].

Caryophyllene, a notable terpenoid, plays a significant role in managing MS-related neuropathic pain and inflammation by selectively binding to CB2 receptors, modulating pain pathways, and exerting anti-inflammatory effects. It offers pain relief without the psychoactive effects of other cannabinoids, making it an alternative to opioid treatments with significant risks of dependence and adverse effects [88]. Limonene, commonly derived from citrus fruits, demonstrates analgesic effects by enhancing serotonin and norepinephrine levels in the CNS, altering pain signal perception in the brain [89,90]. Myrcene, found in cannabis and hops, is known for its sedative and muscle-relaxant properties, potentiating cannabinoid receptor signaling to enhance analgesic effects, particularly in MS patients [88,91]. Pinene also has the potential to improve cognitive function and memory, which benefits chronic pain sufferers who often experience cognitive decline [92].

### 7.3. Alkaloids

Alkaloids, organic compounds containing nitrogen, are found in plants, fungi, and bacteria and include well-known analgesic compounds such as tetrahydropalmatine, matrine, and tetrandrine [93,94]. Capsaicin is particularly effective in managing neuropathic pain by selectively activating the TRPV1 receptor, which initially excites sensory neurons before leading to desensitization. This process depletes neuropeptides such as substance P, reducing pain transmission [95]. Capsaicin’s role in managing neuropathic pain is enhanced by its capacity to alter neuropeptide release in the dorsal horn of the spinal cord, which disrupts pain signaling pathways at both peripheral and central levels [96]. Capsaicin is commonly used in high-concentration topical formulations, such as the 8% capsaicin patch (Qutenza), approved for localized neuropathic pain conditions such as postherpetic neuralgia and diabetic peripheral neuropathy [97,98]. Tetrahydropalmatine (THP) modulates neurotransmitter systems by enhancing dopaminergic and serotonergic activity, contributing to pain relief and improving mood. THP also reduces glutamate release in the CNS, which may attenuate excitotoxicity and neuronal damage, common features of chronic pain states [99]. Matrine has demonstrated neuroprotective effects, particularly in MS, by crossing the blood-brain barrier and supporting myelin restoration while reducing pro-inflammatory cytokine levels. It helps rectify neurotransmitter imbalances, alleviating symptoms such as mechanical allodynia and thermal hyperalgesia without significant adverse effects on motor coordination [100,101].

### 7.4. Other Relevant Phytochemicals

In addition to their anti-inflammatory effects, resveratrol, curcumin, gingerol, and alpha-lipoic acid have been extensively studied for their neuroprotective effects and potential in modulating neurotransmitter activity, enhancing their role in managing neuropathic pain.

Resveratrol and curcumin are both popular natural substances commonly used as dietary supplements [102]. Resveratrol has shown potential in alleviating neuropathic pain through its anti-inflammatory, antioxidant, and neuroprotective effects. Studies suggest it modulates pain pathways by inhibiting pro-inflammatory cytokines and regulating neuroinflammation. In animal models, resveratrol reduced pain sensitivity and improved motor function, likely by interacting with pathways such as NF-κB and Nrf2, which are involved in inflammation and oxidative stress [103]. Resveratrol’s impact on neuroinflammation and oxidative stress plays a significant role in preventing neuronal injury and reducing pain. Although these effects hold promise, more clinical trials in humans are required to confirm the therapeutic potential of resveratrol for neuropathic pain treatment [103].

Curcumin, due to its anti-inflammatory and antioxidant properties, may help alleviate side effects associated with cancer treatments such as chemotherapy and radiotherapy, including reducing gastrointestinal, cardiovascular, kidney, and ototoxicity, as well as easing symptoms such as nausea, vomiting, and loss of appetite [104]. Curcumin inhibits NLRP3 inflammasome activation, modulating both central and peripheral inflammation, which plays a critical role in neuropathic pain management. By reducing this pathway and mitigating GSK-3β activation, it helped reduce pain and improve motor function in mice with neuropathic pain [105]. Additionally, curcumin supports neuronal survival by activating key signaling pathways such as AMPK and sirtuins [106]. Furthermore, curcumin supplementation is safe and well-tolerated, with no adverse effects reported in an adolescent with Déjérine-Sottas disease [107]. Nano-curcumin supplementation (80 mg) has shown positive effects on depression and anxiety symptoms, though its impact on stress levels remains unclear [108].

Ginger has demonstrated antidiabetic and analgesic effects in management of DNP [109]. By reducing inflammation and modulating neuroinflammation, ginger enhances its potential to alleviate pain associated with diabetic neuropathy. Furthermore, by influencing serotonin receptors, it may help improve mood regulation, contributing to its overall analgesic effects in DNP [109,110].

Below, Table 1 presents a summary of the most commonly studied phytochemicals in neuropathic pain, their mechanisms of action, and their effects.

## 8. Clinical Trials of Phytochemicals

Clinical trials evaluating the effectiveness of phytochemicals in managing neuropathic pain have produced mixed results, which vary based on formulation, dosage, and specific patient conditions. As previously mentioned, high-concentration capsaicin patches (8%) have been extensively studied in patients with diabetic neuropathy and postherpetic neuralgia [96,97]. These trials indicate that capsaicin effectively desensitizes nociceptive neurons through the modulation of TRPV1 receptors, providing significant pain relief that can persist for up to 12 weeks after a single application [112]. The side effects are generally limited to localized reactions, making capsaicin a viable option for managing localized neuropathic pain [113].

Similarly, clinical studies involving oral curcumin supplements have shown promising yet variable results in patients with chronic pain syndromes, including neuropathic pain. Many trials report reductions in pain scores and improvements in quality of life, which can be attributed to curcumin’s anti-inflammatory and antioxidant properties [114]. However, challenges related to its low bioavailability have led to the investigation of newer formulations, such as curcumin nanoparticles, to enhance its efficacy [115].

Several studies are also exploring the potential of combining phytochemicals, such as curcumin and resveratrol, with conventional medications to enhance analgesic effects. These combination therapies may leverage synergistic mechanisms, leading to improved pain relief and reduced side effects [116].

Research on resveratrol is more limited, but early-phase clinical trials suggest that it may help alleviate neuropathic symptoms by targeting oxidative stress and inflammatory pathways [117,118]. Small-scale studies indicate modest pain relief and improved patient-reported outcomes, particularly in cases of diabetic neuropathy. However, further trials with larger cohorts are necessary to validate these findings [103,119,120].

Polydatin (PLD) has shown promise in treating neuropathic pain by reducing oxidative stress and inflammation. Studies suggest that PLD enhances antioxidant activity (e.g., catalase and glutathione), reduces nitrite levels, and regulates matrix metalloproteinase (MMP) activity, which helps protect neuronal tissue after spinal cord injury. These effects contribute to pain relief and improved sensory-motor function [121].

Clinical trials also suggest that cannabinoids may provide relief in conditions such as multiple sclerosis-related neuropathy and diabetic neuropathy by modulating inflammatory and oxidative stress pathways. However, results have been mixed, and further research is required to identify the most effective formulations and dosages. Early studies highlight their potential as an adjunct to conventional therapies, but larger, high-quality trials are needed to confirm these findings [2].

It is important to note that research on curcumin and other phytochemicals in the treatment of neuropathic pain is still in its developmental phase. Given the limited clinical trial data, further high-quality studies are needed to establish their clinical efficacy and safety.

## 9. Conclusions

Some phytochemicals have shown promising potential in managing neuropathic pain by targeting mechanisms such as inflammation reduction, alleviating oxidative stress, and modulating pain signaling pathways. These plant-derived compounds offer a lower-risk alternative to conventional treatments, which are often limited by side effects and suboptimal long-term efficacy. However, challenges remain in their routine clinical application, including improving bioavailability, ensuring consistent efficacy, and developing standardized treatment protocols. While phytochemicals are not yet a definitive solution, they represent a significant advancement in neuropathic pain management and hold promise as complementary or alternative therapies. Ongoing research and high-quality clinical trials are essential to validate their therapeutic potential. However, are we on the verge of new solutions? The answer is cautiously optimistic.

This cautious optimism is due to the growing body of evidence that supports the efficacy of certain phytochemicals despite the limitations and gaps in current clinical trials. While it is clear that more research is needed to establish these compounds as mainstream treatments, their ability to offer a lower-risk alternative to conventional medications presents a compelling reason to continue exploring their full potential.

## Figures and Tables

**Table 1:** Mechanisms of action of phytochemicals in neuropathic pain management.

Phytochemical	Mechanism of Action	References	Structural Formula	Category	Type of Study	Study Results
Narirutin	Selectively inhibits Nav1.7 voltage-gated sodium channels	[79]	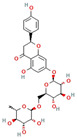	Flavonoid	In vivo	Mechanical withdrawal threshold: 10.5 ± 0.8 g (vs. 7.3 ± 0.5 g in control) Thermal withdrawal latency: 13.2 ± 0.5 s (vs. 10.6 ± 0.4 s in control) *p* < 0.05
Diosmin	Reduces inflammation (NF-κB, TNF-α, COX-2), alleviates neuropathic pain via NO/cGMP/PKG/KATP pathway and spinal cytokine inhibition (IL-1β)	[80,81]	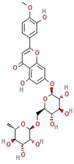	Flavonoid	In vivo	Inflammatory Markers: RF, TNF-α, ACPA, IL-17 decreased by 77%, 65%, 67%, and 72%, respectively; Oxidative Stress: LPO decreased by 38%; Western Blot: NF-κB p50/p65 down by 45%/38%, iNOS by 46%, Nrf2 up by 224%
Quercetin	Antioxidant, anti-inflammatory, modulates immune responses	[82,83]	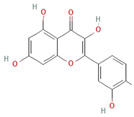	Flavonoid	In vivo	Reduced Bax/Bcl-2 ratio, reduced Cyto. c expression; Caspase-3 activity reduced in cortex and hippocampus (*p* < 0.05); 8 mice/group for western blot, 5 mice/group for confocal microscopy; enhanced neuronal survival (*p* < 0.05)
6-Methoxyflavanone (6-MeOF)	Interacts with GABA-ergic and opioidergic systems	[6]	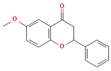	Flavonoid	In vivo	Significant attenuation of nociception at 10 and 30 mg/kg after 30 and 60 min
Berberine	Modulates glucose metabolism, inflammation, and lipid levels	[84]	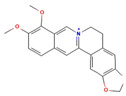	Flavonoid	In vivo	Effect on Body Weight: 211 ± 0.8 g (20 mg/kg) and 250 ± 1.4 g (40 mg/kg) vs. Diabetic control (161 ± 1.0 g) Fasting Blood Glucose: Reduction in glucose (*p* < 0.001) vs. Diabetic control (421 ± 2.0 mg/dL) Serum Insulin Level: Increased to 11.73 ± 0.18 μIU/mL (20 mg/kg and 40 mg/kg) vs. Diabetic control (6.773 ± 0.07 μIU/mL) Thermal Hyperalgesia: Increased pain threshold (dose-dependent, *p* < 0.001) Mechanical Hyperalgesia: Increased pain threshold (dose-dependent, *p* < 0.001) Antioxidant Enzymes (GSH and SOD): GSH: 0.51 ± 0.02 μM/mg protein, SOD: 15.96 ± 0.15 U/mg protein Lipid Peroxidation (TBARS): Reduced to 3.16 ± 0.069 nmol/mg protein (vs. Diabetic control: 6.53 ± 0.15 nmol/mg protein) AGEs (Advanced Glycation End-products): Reduced to 2.48 ± 0.02 RFU/mg protein (vs. Diabetic control: 3.72 ± 0.02 RFU/mg protein) Nitrite Level: Significant reduction, but Gabapentin more effective
Alpha-Tocopherol	Antioxidant, protects cell membranes, modulates immune responses	[84]	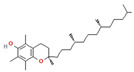	Flavonoid	In vivo	Effect on Body Weight: 250 ± 1.4 g (1000 mg/kg) vs. Diabetic control (161 ± 1.0 g) Fasting Blood Glucose: Reduction in glucose (dose-dependent, *p* < 0.001) Serum Insulin Level: Increased to 11.73 ± 0.18 μIU/mL (20 mg/kg and 40 mg/kg) Thermal Hyperalgesia: Increased pain threshold (dose-dependent) Mechanical Hyperalgesia: Increased pain threshold Antioxidant Enzymes (GSH and SOD): GSH: 0.51 ± 0.02 μM/mg protein, SOD: 15.96 ± 0.15 U/mg protein Lipid Peroxidation (TBARS): Reduced to 3.16 ± 0.069 nmol/mg protein AGEs (Advanced Glycation End-products): Reduced to 2.48 ± 0.02 RFU/mg protein Nitrite Level: Significant reduction (dose-dependent)
Caryophyllene	Binds to CB2 receptors, modulates pain pathways	[88]	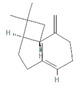	Terpenoid	In vivo	- BCP (1–100 μM) significantly increased IL-10 and decreased IFN-γ production - No change in IL-4 levels after MOG35–55 stimulation - CB2 selective antagonist AM630 (50 μM) blocked BCP’s immunomodulatory effect. - In EAE model, clinical score peaked at 3.5 on day 19 post-immunization - 25 mg/kg BCP reduced motor paralysis and weight loss. - BCP (50 mg/kg) significantly reduced mechanical hyperalgesia
Limonene	Interacts with serotonin and norepinephrine systems	[89,90]	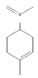	Terpenoid	In vivo	BDNF: Decreased in Str (significantly lower than C, *p* < 0.05), increased in Str + Lim (higher than Str). IL-1β: 5.33 ± 0.42 (Str), 3.16 ± 0.41 (C), 2.85 ± 0.24 (Lim), 4.07 ± 0.1 (Str + Lim) − significantly reduced in Str + Lim compared to Str. Caspase-1: 0.55 ± 0.06 (Str + Lim), 0.32 ± 0.04 (Lim) − significantly higher in Str + Lim than Lim, *p* = 0.009. IL-6: No significant differences (*p* > 0.05).
Mycrene	Modulates cannabinoid receptors	[88,91]	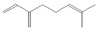	Terpenoid	In vivo	- Nociception (secondary allodynia): 1 mg/kg dose improved nociception by 211.0 ± 17.93%; 5 mg/kg dose improved nociception by 269.3 ± 63.27%. - Blockade of CB receptors: CB1 antagonist AM281 blocked myrcene’s analgesic effect (*p* < 0.001), CB2 antagonist AM630 blocked it (*p* < 0.0001). - Leukocyte Rolling: Myrcene reduced leukocyte rolling at 60 min (*p* < 0.0001). - CB2 Antagonist Blockade: AM630 reduced leukocyte rolling (*p* < 0.05). - Chronic Pain: Repeated myrcene administration increased paw withdrawal threshold (*p* < 0.0001).
Pinene	Modulates cannabinoid receptors	[92]	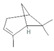	Terpenoid	In vivo	IL-1β (skin): α-Pinene 1 mg/kg: 62.68 ± 4.54 α-Pinene 5 mg/kg: 45.74 ± 1.48 α-Pinene 10 mg/kg: 47.75 ± 4.44 TNF-α (skin): α-Pinene 1 mg/kg: 92.02 ± 4.84 α-Pinene 5 mg/kg: 56.36 ± 6.02 α-Pinene 10 mg/kg: 61.23 ± 3.25 SOD (skin): α-Pinene 1 mg/kg: 27.91 ± 2.88 α-Pinene 5 mg/kg: 41.49 ± 1.75 α-Pinene 10 mg/kg: 47.42 ± 3.02
Capsaicin	Desensitizes TRPV1 receptors, modulates pain signaling	[95,96,97]	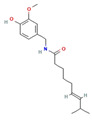	Alkaloid	In vivo	Axon reflex flare abolished during capsaicin, recovered to ~50% after 49 days. All sensations recovered completely within 7 weeks in healthy subjects. Analgesia lasted for months in spontaneous neuropathic pain patients treated with 8% capsaicin.
Tetrahydropalmatine (THP)	Modulates dopaminergic and serotonergic activity, decreases glutamate release	[98]	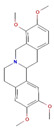	Alkaloid	In vivo In vitro	- THP (5 mg/kg, 10 mg/kg) alleviates mechanical allodynia and heat hyperalgesia in CFA-induced inflammatory pain rats (observed on Day 9) - 2.5 mg/kg did not significantly relieve pain - Gait parameters: THP treatment significantly reversed CFA-induced reductions in contact area and print length (Day 7) - 100 μM THP promoted significant apoptosis in astrocytes and microglia - 10 mg/kg THP reduced spinal cord inflammatory cytokines (TNF-α, IL-1β) and NF-κB activation - Significant reduction in p-NF-κB/NF-κB ratio after THP treatment
Matrine	Modulates neurotransmitter imbalances supports myelin restoration, and reduces pro-inflammatory cytokines	[99,100]	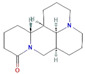	Alkaloid	In vivo	Paw withdrawal threshold: 0.88 ± 0.16 (Matrine) vs 0.18 ± 0.04 (CCI) Paw withdrawal latency: 7.01 ± 0.11 (Matrine) vs 4.62 ± 0.18 (CCI) Counts of paw withdrawal: 19.7 ± 1.15 (Matrine) vs 44.3 ± 2.99 (CCI)
Resveratrol	Modulates pro-inflammatory cytokines, oxidative stress, and neuroinflammatory responses	[101,102]	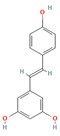	Phenolic Compound	In vivo	40 mg/kg reduced thermal hyperalgesia and allodynia significantly. No effect at 5 mg/kg. Cytokine modulation: TNF-α, IL-1β, IL-6 decreased, IL-10 increased in a dose-dependent manner. Significant inhibition of TNF-α, IL-1β, IL-6 at 1, 2, and 5 μM. IL-10 secretion promoted. NO level reduced in Aβ-stimulated microglia. Significant pain relief from day 7 to day 21 after CCI, with maximal effect at day 21.
Curcumin	Modulates inflammation, oxidative stress, and ion channels	[103,104,105,106,107]	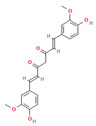	Phenolic Compound	In vivo	Cognitive Impairment (NOR Test): 3.16-fold and 2.07-fold increase in discrimination and preference indices (Curcumin) vs. 128% and 56.49% decrease (EAE) (*p* < 0.0001) Hippocampal Neurons (H and E Staining): 3.06-fold increase in intact neurons (Curcumin) vs. 69.42% decrease (EAE) (*p* < 0.0001) Motor Function: 3.33-, 2.40-, 2.36-, and 3.09-fold increase in distance traveled, mean speed, ambulation, and rearing frequencies (Curcumin) vs. 77.17%, 69.08%, 65.36%, and 76.77% decrease (EAE) (*p* < 0.0001) Protein Levels (AMPK/SIRT1 Pathway): 2.93-fold and 2.77-fold increase in p-AMPKThr172 and SIRT1 (Curcumin) vs. 71.67% and 69.09% decrease (EAE) (*p* < 0.0001) Demyelination (CREB/BDNF/MBP Pathway): 3.35-, 1.84-, and 2.21-fold increase in p-CREBSer133, BDNF, and MBP (Curcumin) vs. 73.27%, 51.90%, and 67.09% decrease (EAE) (*p* < 0.0001)

The structural formulas for all compounds in the table are sourced from PubChem (National Center for Biotechnology Information, 2024). Accessed 3 December 2024. Available online: https://pubchem.ncbi.nlm.nih.gov/. Specifically, for each compound, the following PubChem IDs were used: Narirutin (CID 442431), Diosmin (CID 5281613), Quercetin (CID 5280343), 6-Methoxyflavanone (6-MeOF) (CID 147157), Berberine (CID 2353), Tocopherol (CID 14985), Caryophyllene (CID 5281515), Limonene (CID 22311), Myrcene (CID 31253), Pinene (CID 6654), Capsaicin (CID 1548943), Tetrahydropalmatine (THP) (CID 5417), Matrine (CID 91466), Resveratrol (CID 445154), Curcumin (CID 969516), Gingerol (CID 443793), Alpha-lipoic acid (CID 864) [111].

## References

[1] Doneddu P.E., Pensato U., Iorfida A., Alberti C., Nobile-Orazio E., Fabbri A., Voza A., Study and Research Center of the Italian Society of Emergency Medicine (SIMEU) (2023). Neuropathic Pain in the Emergency Setting: Diagnosis and Management. J. Clin. Med..

[2] Cavalli E., Mammana S., Nicoletti F., Bramanti P., Mazzon E. (2019). The Neuropathic Pain: An Overview of the Current Treatment and Future Therapeutic Approaches. Int. J. Immunopathol. Pharmacol..

[3] Woo A.K. (2010). Depression and Anxiety in Pain. Rev. Pain.

[4] Alorfi N.M. (2023). Pharmacological Methods of Pain Management: Narrative Review of Medication Used. Int. J. Gen. Med..

[5] Oveissi V., Ram M., Bahramsoltani R., Ebrahimi F., Rahimi R., Naseri R., Belwal T., Devkota H.P., Abbasabadi Z., Farzaei M.H. (2019). Medicinal Plants and Their Isolated Phytochemicals for the Management of Chemotherapy-Induced Neuropathy: Therapeutic Targets and Clinical Perspective. Daru.

[6] Akbar S., Subhan F., Karim N., Shahid M., Ahmad N., Ali G., Mahmood W., Fawad K. (2016). 6-Methoxyflavanone Attenuates Mechanical Allodynia and Vulvodynia in the Streptozotocin-Induced Diabetic Neuropathic Pain. Biomed. Pharmacother..

[7] Takeda M., Sashide Y., Toyota R., Ito H. (2024). The Phytochemical, Quercetin, Attenuates Nociceptive and Pathological Pain: Neurophysiological Mechanisms and Therapeutic Potential. Molecules.

[8] Balakrishnan R., Azam S., Cho D.Y., Su-Kim I., Choi D.K. (2021). Natural Phytochemicals as Novel Therapeutic Strategies to Prevent and Treat Parkinson’s Disease: Current Knowledge and Future Perspectives. Oxid. Med. Cell. Longev..

[9] Basu P., Maier C., Basu A. (2021). Effects of Curcumin and Its Different Formulations in Preclinical and Clinical Studies of Peripheral Neuropathic and Postoperative Pain: A Comprehensive Review. Int. J. Mol. Sci..

[10] Kumar A., Nirmal P., Kumar M., Jose A., Tomer V., Oz E., Proestos C., Zeng M., Elobeid T., Sneha K. (2023). Major Phytochemicals: Recent Advances in Health Benefits and Extraction Method. Molecules.

[11] Rabizadeh F., Mirian M.S., Doosti R., Kiani-Anbouhi R., Eftekhari E. (2022). Phytochemical Classification of Medicinal Plants Used in the Treatment of Kidney Disease Based on Traditional Persian Medicine. Evid. Based Complement. Alternat. Med..

[12] Paul J.K., Azmal M., Newaz Been Haque A.S., Talukder O.F., Meem M., Ghosh A. (2024). Phytochemical-Mediated Modulation of Signaling Pathways: A Promising Avenue for Drug Discovery. Adv. Redox Res..

[13] Roy A., Khan A., Ahmad I., Alghamdi S., Rajab B.S., Babalghith A.O., Alshahrani M.Y., Islam S., Islam M.R. (2022). Flavonoids: A Bioactive Compound from Medicinal Plants and Its Therapeutic Applications. Biomed. Res. Int..

[14] Baskozos G., Hébert H.L., Pascal M.M., Themistocleous A.C., Macfarlane G.J., Wynick D., Bennett D.L., Smith B.H. (2023). Epidemiology of Neuropathic Pain: An Analysis of Prevalence and Associated Factors in UK Biobank. Pain Rep..

[15] van Hecke O., Austin S.K., Khan R.A., Smith B.H., Torrance N. (2014). Neuropathic Pain in the General Population: A Systematic Review of Epidemiological Studies. Pain.

[16] Mitsikostas D.D., Moka E., Orrillo E., Aurilio C., Vadalouca A., Paladini A., Varrassi G. (2022). Neuropathic Pain in Neurologic Disorders: A Narrative Review. Cureus.

[17] Feldman E.L., Callaghan B.C., Pop-Busui R., Zochodne D.W., Wright D.E., Bennett D.L., Bril V., Russell J.W., Viswanathan V. (2019). Diabetic Neuropathy. Nat. Rev. Dis. Primers.

[18] Pop-Busui R., Ang L., Boulton AJ M., Feldman E.L., Marcus R.L., Mizokami-Stout K., Ziegler D. (2022). Diagnosis and Treatment of Painful Diabetic Peripheral Neuropathy.

[19] Machado-Duque M.E., Gaviria-Mendoza A., Machado-Alba J.E., Castaño N. (2020). Evaluation of Treatment Patterns and Direct Costs Associated with the Management of Neuropathic Pain. Pain Res Manag..

[20] Schaefer C., Sadosky A., Mann R., Daniel S., Parsons B., Tuchman M., Anschel A., Stacey B.R., Nalamachu S., Nieshoff E. (2014). Pain Severity and the Economic Burden of Neuropathic Pain in the United States: BEAT Neuropathic Pain Observational Study. Clinicoecon Outcomes Res..

[21] Colloca L., Ludman T., Bouhassira D., Baron R., Dickenson A.H., Yarnitsky D., Freeman R., Truini A., Attal N., Finnerup N.B. (2017). Neuropathic pain. Nat. Rev. Dis. Primers.

[22] Nieto F.R., Vuckovic S.M., Prostran M.S. (2020). Editorial: Mechanisms and New Targets for the Treatment of Chronic Pain. Front. Pharmacol..

[23] Chen X., Pang R.-P., Shen K.-F., Zimmermann M., Xin W.-J., Li Y.-Y., Liu X.-G. (2011). TNF-α Enhances the Currents of Voltage-Gated Sodium Channels in Uninjured Dorsal Root Ganglion Neurons Following Motor Nerve Injury. Exp. Neurol..

[24] Duan Y.-W., Chen S.-X., Li Q.-Y., Zang Y. (2022). Neuroimmune Mechanisms Underlying Neuropathic Pain: The Potential Role of TNF-α-Necroptosis Pathway. Int. J. Mol. Sci..

[25] Karavis M.Y., Siafaka I., Vadalouca A., Georgoudis G. (2023). Role of Microglia in Neuropathic Pain. Cureus.

[26] Detloff M.R., Fisher L.C., McGaughy V., Longbrake E.E., Popovich P.G., Basso D.M. (2008). Remote Activation of Microglia and Pro-Inflammatory Cytokines Predict the Onset and Severity of Below-Level Neuropathic Pain after Spinal Cord Injury in Rats. Exp. Neurol..

[27] Wang C., Gu L., Ruan Y., Gegen T., Yu L., Zhu C., Yang Y., Zhou Y., Yu G., Tang Z. (2018). Pirt Together with TRPV1 Is Involved in the Regulation of Neuropathic Pain. Neural Plast..

[28] Wen B., Pan Y., Cheng J., Xu L., Xu J. (2023). The Role of Neuroinflammation in Complex Regional Pain Syndrome: A Comprehensive Review. J. Pain Res..

[29] Ilari S., Giancotti L.A., Lauro F., Gliozzi M., Malafoglia V., Palma E., Tafani M., Russo M.A., Tomino C., Fini M. (2020). Natural Antioxidant Control of Neuropathic Pain—Exploring the Role of Mitochondrial SIRT3 Pathway. Antioxidants.

[30] Olufunmilayo E.O., Gerke-Duncan M.B., Holsinger R.M.D. (2023). Oxidative Stress and Antioxidants in Neurodegenerative Disorders. Antioxidants.

[31] Silva Santos Ribeiro P., Willemen H.L.D.M., Versteeg S., Gil C.M., Eijkelkamp N. (2023). NLRP3 Inflammasome Activation in Sensory Neurons Promotes Chronic Inflammatory and Osteoarthritis Pain. Immunother. Adv..

[32] Kirkley K.S., Popichak K.A., Afzali M.F., Legare M.E., Tjalkens R.B. (2017). Microglia Amplify Inflammatory Activation of Astrocytes in Manganese Neurotoxicity. J. Neuroinflamm..

[33] Ashok A., Andrabi S.S., Mansoor S., Kuang Y., Kwon B.K., Labhasetwar V. (2022). Antioxidant Therapy in Oxidative Stress-Induced Neurodegenerative Diseases: Role of Nanoparticle-Based Drug Delivery Systems in Clinical Translation. Antioxidants.

[34] Yam M.F., Loh Y.C., Tan C.S., Khadijah Adam S., Abdul Manan N., Basir R. (2018). General Pathways of Pain Sensation and the Major Neurotransmitters Involved in Pain Regulation. Int. J. Mol. Sci..

[35] Kendroud S., Fitzgerald L.A., Murray I.V., Hanna A. (2024). Physiology, Nociceptive Pathways. StatPearls.

[36] Al-Chalabi M., Reddy V., Gupta S. (2024). Neuroanatomy, Spinothalamic Tract. StatPearls.

[37] Ji R.R., Nackley A., Huh Y., Terrando N., Maixner W. (2018). Neuroinflammation and Central Sensitization in Chronic and Widespread Pain. Anesthesiology.

[38] McBenedict B., Goh K.S., Yau R.C.C., Elamin S., Yusuf W.H., Verly G., Thomas A., Alphonse B., Ouabicha K., Valentim G. (2024). Neuropathic Pain Secondary to Multiple Sclerosis: A Narrative Review. Cureus.

[39] Bodman M.A., Dreyer M.A., Varacallo M. (2024). Diabetic Peripheral Neuropathy. StatPearls.

[40] Basta I., Bozovic I., Berisavac I., Stojiljkovic-Tamas O., Rajic S.L., Dominovic-Kovacevic A., Stojanov A., Djordjevic G., Jovanovic D., Peric S. (2019). Recurrent Guillain-Barré Syndrome—Case Series. Neurol. India.

[41] Leonhard S.E., Mandarakas M.R., Gondim F.A.A., Bateman K., Ferreira M.L.B., Cornblath D.R., van Doorn P.A., Dourado M.E., Hughes R.A.C., Islam B. (2019). Diagnosis and Management of Guillain-Barré Syndrome in Ten Steps. Nat. Rev. Neurol..

[42] Shastri A., Al Aiyan A., Kishore U., Farrugia M.E. (2023). Immune-Mediated Neuropathies: Pathophysiology and Management. Int. J. Mol. Sci..

[43] Vukojevic Z., Berisavac I., Bozovic I., Dominovic-Kovacevic A., Lavrnic D., Peric S. (2021). Longitudinal Study of Neuropathic Pain in Patients with Guillain-Barré Syndrome. Ir. J. Med. Sci..

[44] Bellanti R., Rinaldi S. (2024). Guillain-Barré Syndrome: A Comprehensive Review. Eur. J. Neurol..

[45] Hagen K.M., Ousman S.S. (2021). The Neuroimmunology of Guillain-Barré Syndrome and the Potential Role of an Aging Immune System. Front. Aging Neurosci..

[46] Dimachkie M.M., Barohn R.J. (2013). Guillain-Barré Syndrome and Variants. Neurol. Clin..

[47] Deer T.R., Eldabe S., Falowski S.M., Huntoon M.A., Staats P.S., Cassar I.R., Crosby N.D., Boggs J.W. (2021). Peripherally Induced Reconditioning of the Central Nervous System: A Proposed Mechanistic Theory for Sustained Relief of Chronic Pain with Percutaneous Peripheral Nerve Stimulation. J. Pain Res..

[48] Ma R.S.Y., Kayani K., Whyte-Oshodi D., Whyte-Oshodi A., Nachiappan N., Gnanarajah S., Mohammed R. (2019). Voltage Gated Sodium Channels as Therapeutic Targets for Chronic Pain. J. Pain Res..

[49] Yao S., Chen H., Zhang Q., Shi Z., Liu J., Lian Z., Feng H., Du Q., Xie J., Ge W. (2018). Pain during the acute phase of Guillain-Barré syndrome. Medicine.

[50] Swami T., Khanna M., Gupta A., Prakash N.B. (2021). Neuropathic Pain in Guillain-Barre Syndrome: Association with Rehabilitation Outcomes and Quality of Life. Ann. Indian Acad. Neurol..

[51] Hillyar C., Nibber A. (2020). Psychiatric Sequelae of Guillain-Barré Syndrome: Towards a Multidisciplinary Team Approach. Cureus.

[52] Jovanović A., Pekmezović T., Mesaros S., Novaković I., Peterlin B., Veselinović N., Tamas O., Ivanović J., Marić G., Andabaka M. (2024). Exclusive Breastfeeding May Be a Protective Factor in Individuals with Familial Multiple Sclerosis: A Population Registry-Based Case-Control Study. Mult. Scler. Relat. Disord..

[53] Gómez-Melero S., Caballero-Villarraso J., Escribano B.M., Galvao-Carmona A., Túnez I., Agüera-Morales E. (2024). Impact of Cognitive Impairment on Quality of Life in Multiple Sclerosis Patients: A Comprehensive Review. J. Clin. Med..

[54] Racke M.K., Frohman E.M., Frohman T. (2022). Pain in Multiple Sclerosis: Understanding Pathophysiology, Diagnosis, and Management Through Clinical Vignettes. Front. Neurol..

[55] Maric G., Pekmezovic T., Tamas O., Veselinovic N., Jovanovic A., Lalic K., Mesaros S., Drulovic J. (2022). Impact of Comorbidities on the Disability Progression in Multiple Sclerosis. Acta Neurol. Scand..

[56] Drulovic J., Pekmezovic T., Tamas O., Adamec I., Aleksic D., Andabaka M., Basic Kes V., Butkovic Soldo S., Cukic M., Despinic L. (2023). The Impact of the Comorbid Seizure/Epilepsy on the Health-Related Quality of Life in People with Multiple Sclerosis: An International Multicentric Study. Front. Immunol..

[57] Murphy K.L., Bethea J.R., Fischer R., Zagon I.S., McLaughlin P.J. (2017). Neuropathic Pain in Multiple Sclerosis—Current Therapeutic Intervention and Future Treatment Perspectives. Multiple Sclerosis: Perspectives in Treatment and Pathogenesis.

[58] Coggan J.S., Bittner S., Stiefel K.M., Meuth S.G., Prescott S.A. (2015). Physiological Dynamics in Demyelinating Diseases: Unraveling Complex Relationships through Computer Modeling. Int. J. Mol. Sci..

[59] Bokan-Mirković V., Škarić-Karanikić Ž., Nejkov S., Vuković M., Ćirović D. (2017). Diabetic Polyneuropathy and Risk of Falls: Fear of Falling and Other Factors. Acta Clin. Croat..

[60] Syed O., Jancic P., Knezevic N.N. (2023). A Review of Recent Pharmacological Advances in the Management of Diabetes-Associated Peripheral Neuropathy. Pharmaceuticals.

[61] Darenskaya M., Kolesnikov S., Semenova N., Kolesnikova L. (2023). Diabetic Nephropathy: Significance of Determining Oxidative Stress and Opportunities for Antioxidant Therapies. Int. J. Mol. Sci..

[62] Smith S., Normahani P., Lane T., Hohenschurz-Schmidt D., Oliver N., Davies A.H. (2022). Prevention and Management Strategies for Diabetic Neuropathy. Life.

[63] Singh V.P., Bali A., Singh N., Jaggi A.S. (2014). Advanced Glycation End Products and Diabetic Complications. Korean J. Physiol. Pharmacol..

[64] Caturano A., D’Angelo M., Mormone A., Russo V., Mollica M.P., Salvatore T., Galiero R., Rinaldi L., Vetrano E., Marfella R. (2023). Oxidative Stress in Type 2 Diabetes: Impacts from Pathogenesis to Lifestyle Modifications. Curr. Issues Mol. Biol..

[65] Wiffen P.J., Derry S., Moore R.A., Aldington D., Cole P., Rice A.S., Lunn M.P., Hamunen K., Haanpaa M., Kalso E.A. (2013). Antiepileptic drugs for neuropathic pain and fibromyalgia—An overview of Cochrane reviews. Cochrane Database Syst. Rev..

[66] Moore R.A., Derry S., Aldington D., Cole P., Wiffen P.J. (2015). Amitriptyline for neuropathic pain in adults. Cochrane Database Syst. Rev..

[67] Gilron I., Tu D., Holden R.R., Jackson A.C., DuMerton-Shore D. (2015). Combination of Morphine with Nortriptyline for Neuropathic Pain. Pain.

[68] Lunn M.P., Hughes R.A., Wiffen P.J. (2014). Duloxetine for treating painful neuropathy, chronic pain or fibromyalgia. Cochrane Database Syst. Rev..

[69] Duehmke R.M., Derry S., Wiffen P.J., Bell R.F., Aldington D., Moore R.A. (2017). Tramadol for neuropathic pain in adults. Cochrane Database Syst. Rev..

[70] McNicol E.D., Midbari A., Eisenberg E. (2013). Opioids for neuropathic pain. Cochrane Database Syst. Rev..

[71] Liu J., Wang L.N., McNicol E.D. (2015). Pharmacological treatment for pain in Guillain-Barré syndrome. Cochrane Database Syst Rev..

[72] Alothman L., Alhadlaq E., Alhussain A., Alabdulkarim A., Sari Y., AlSharari S.D. (2024). New Pharmacological Insight into Etanercept and Pregabalin in Allodynia and Nociception: Behavioral Studies in a Murine Neuropathic Pain Model. Brain Sci..

[73] Gawande I., Akhuj A., Samal S. (2024). Effectiveness of Physiotherapy Intervention in Guillain-Barré Syndrome: A Case Report. Cureus.

[74] Evancho A., Tyler W.J., McGregor K. (2023). A Review of Combined Neuromodulation and Physical Therapy Interventions for Enhanced Neurorehabilitation. Front. Hum. Neurosci..

[75] Kluding P.M., Bareiss S.K., Hastings M., Marcus L.R., Sinacore D.R., Mueller M.J. (2017). Physical Training and Activity in People with Diabetic Peripheral Neuropathy: Paradigm Shift. Phys. Ther..

[76] Ahmad F., Joshi S.H. (2023). Self-Care Practices and Their Role in the Control of Diabetes: A Narrative Review. Cureus.

[77] Paunovic V., Peric S., Vukovic I., Stamenkovic M., Milosevic E., Stevanovic D., Mandic M., Basta I., Berisavac I., Arsenijevic M. (2022). Downregulation of LKB1/AMPK Signaling in Blood Mononuclear Cells Is Associated with the Severity of Guillain–Barre Syndrome. Cells.

[78] Panche A.N., Diwan A.D., Chandra S.R. (2016). Flavonoids: An Overview. J. Nutr. Sci..

[79] Yang H., Shan Z., Guo W., Wang Y., Cai S., Li F., Huang Q., Liu J.A., Cheung C.W., Cai S. (2022). Reversal of Peripheral Neuropathic Pain by the Small-Molecule Natural Product Narirutin via Block of Na_v_1.7 Voltage-Gated Sodium Channel. Int. J. Mol. Sci..

[80] Shaaban H.H., Hozayen W.G., Khaliefa A.K., El-Kenawy A.E., Ali T.M., Ahmed O.M. (2022). Diosmin and Trolox Have Anti-Arthritic, Anti-Inflammatory and Antioxidant Potencies in Complete Freund’s Adjuvant-Induced Arthritic Male Wistar Rats: Roles of NF-κB, iNOS, Nrf2 and MMPs. Antioxidants.

[81] Carballo-Villalobos A.I., González-Trujano M.E., Pellicer F., Alvarado-Vásquez N., López-Muñoz F.J. (2018). Central and Peripheral Anti-Hyperalgesic Effects of Diosmin in a Neuropathic Pain Model in Rats. Biomed. Pharmacother..

[82] Anjaneyulu M., Chopra K. (2003). Quercetin, a bioflavonoid, attenuates thermal hyperalgesia in a mouse model of diabetic neuropathic pain. Prog. Neuropsychopharmacol. Biol. Psychiatry.

[83] Khan A., Ali T., Rehman S.U., Khan M.S., Alam S.I., Ikram M., Muhammad T., Saeed K., Badshah H., Kim M.O. (2018). Neuro-protective Effect of Quercetin Against the Detrimental Effects of LPS in the Adult Mouse Brain. Front. Pharmacol..

[84] Alkholifi F.K., Aodah A.H., Foudah A.I., Alam A. (2023). Exploring the Therapeutic Potential of Berberine and Tocopherol in Managing Diabetic Neuropathy: A Comprehensive Approach towards Alleviating Chronic Neuropathic Pain. Biomedicines.

[85] Del Prado-Audelo M.L., Cortés H., Caballero-Florán I.H., González-Torres M., Escutia-Guadarrama L., Bernal-Chávez S.A., Giraldo-Gomez D.M., Magaña J.J., Leyva-Gómez G. (2021). Therapeutic Applications of Terpenes on Inflammatory Diseases. Front. Pharmacol..

[86] Bie B., Wu J., Foss J.F., Naguib M. (2018). An overview of the cannabinoid type 2 receptor system and its therapeutic potential. Curr. Opin. Anaesthesiol..

[87] Liktor-Busa E., Keresztes A., LaVigne J., Streicher J.M., Largent-Milnes T.M., Barker E. (2021). Analgesic Potential of Terpenes Derived from *Cannabis sativa*. Pharmacol. Rev..

[88] Alberti T.B., Barbosa W.L.R., Vieira J.L.F., Raposo N.R.B., Dutra R.C. (2017). (−)-β-Caryophyllene, a CB2 Receptor-Selective Phytocannabinoid, Suppresses Motor Paralysis and Neuroinflammation in a Murine Model of Multiple Sclerosis. Int. J. Mol. Sci..

[89] Alkanat M., Alkanat H.Ö. (2024). D-Limonene Reduces Depression-Like Behaviour and Enhances Learning and Memory through an Anti-Neuroinflammatory Mechanism in Male Rats Subjected to Chronic Restraint Stress. Eur. J. Neurosci..

[90] Eddin L.B., Jha N.K., Meeran M.F.N., Kesari K.K., Beiram R., Ojha S. (2021). Neuroprotective Potential of Limonene and Limonene Containing Natural Products. Molecules.

[91] McDougall J.J., McKenna M.K. (2022). Anti-Inflammatory and Analgesic Properties of the Cannabis Terpene Myrcene in Rat Adjuvant Monoarthritis. Int. J. Mol. Sci..

[92] Rahimi K., Zalaghi M., Shehnizad E.G., Salari G., Baghdezfoli F., Ebrahimifar A. (2023). The Effects of Alpha-Pinene on Inflammatory Responses and Oxidative Stress in the Formalin Test. Brain Res. Bull..

[93] Qiu S., Sun H., Zhang A.-H., Xu H.-Y., Yan G.-L., Han Y., Wang X.-J. (2014). Natural Alkaloids: Basic Aspects, Biological Roles, and Future Perspectives. Chin. J. Nat. Med..

[94] Jiang W., Tang M., Yang L., Zhao X., Gao J., Jiao Y., Li T., Tie C., Gao T., Han Y. (2022). Analgesic Alkaloids Derived from Traditional Chinese Medicine in Pain Management. Front. Pharmacol..

[95] Gualdani R., Barbeau S., Yuan J.H., Jacobs D.S., Gailly P., Dib-Hajj S.D., Waxman S.G. (2024). TRPV1 Corneal Neuralgia Mutation: Enhanced pH Response, Bradykinin Sensitization, and Capsaicin Desensitization. Proc. Natl. Acad. Sci. USA.

[96] Peppin J.F., Pappagallo M. (2014). Capsaicinoids in the Treatment of Neuropathic Pain: A Review. Ther. Adv. Neurol. Disord..

[97] Tailliez N., Planche L., Dorion A., Kacki N., Dimet J., Pluchon Y.M. (2024). Effect of Cooling Capsaicin Application Site on Reducing Burning Sensation in Neuropathic Pain Patients: A Randomized Controlled Trial. Pain Manag. Nurs..

[98] Tumbala Gutti D., Carr R., Schmelz M., Rukwied R. (2024). Slow Depolarizing Electrical Stimuli Reveal Differential Time Courses of Nociceptor Recovery after Prolonged Topical Capsaicin in Human Skin. Eur. J. Pain.

[99] Liu B., Luo M., Meng D., Pan H., Shen H., Shen J., Yao M., Xu L. (2021). Tetrahydropalmatine Exerts Analgesic Effects by Promoting Apoptosis and Inhibiting the Activation of Glial Cells in Rats with Inflammatory Pain. Mol. Pain.

[100] Chhabra S., Mehan S. (2023). Matrine Exerts Its Neuroprotective Effects by Modulating Multiple Neuronal Pathways. Metab. Brain Dis..

[101] Haiyan W., Yuxiang L., Linglu D., Tingting X., Yinju H., Hongyan L., Jianqiang Y. (2013). Antinociceptive Effects of Matrine on Neuropathic Pain Induced by Chronic Constriction Injury. Pharmaceutical Biology.

[102] Cione E., La Torre C., Cannataro R., Caroleo M.C., Plastina P., Gallelli L. (2020). Quercetin, Epigallocatechin Gallate, Curcumin, and Resveratrol: From Dietary Sources to Human MicroRNA Modulation. Molecules.

[103] Tao L., Ding Q., Gao C., Sun X. (2016). Resveratrol Attenuates Neuropathic Pain through Balancing Pro-inflammatory and Anti-inflammatory Cytokines Release in Mice. Int. Immunopharmacol..

[104] Belcaro G., Hosoi M., Pellegrini L., Appendino G., Ippolito E., Ricci A., Ledda A., Dugall M., Cesarone M.R., Maione C. (2014). A controlled study of a lecithinized delivery system of curcumin (Meriva^®^) to alleviate the adverse effects of cancer treatment. Phytother. Res..

[105] Zhang M.W., Sun X., Xu Y.W., Meng W., Tang Q., Gao H., Liu L., Chen S.H. (2024). Curcumin Relieves Oxaliplatin-Induced Neuropathic Pain via Reducing Inflammation and Activating Antioxidant Response. Cell Biol. Int..

[106] Sadek M.A., Rabie M.A., El Sayed N.S., Sayed H.M., Kandil E.A. (2024). Neuroprotective effect of curcumin against experimental autoimmune encephalomyelitis-induced cognitive and physical impairments in mice: An insight into the role of the AMPK/SIRT1 pathway. Inflammopharmacol.

[107] Burns J., Joseph P.D., Rose K.J., Ryan M.M., Ouvrier R.A. (2009). Effect of oral curcumin on Déjérine-Sottas disease. Pediatr. Neurol..

[108] Asadi S., Gholami M.S., Siassi F., Qorbani M., Sotoudeh G. (2020). Beneficial effects of nano-curcumin supplement on depression and anxiety in diabetic patients with peripheral neuropathy: A randomized, double-blind, placebo-controlled clinical trial. Phytother. Res..

[109] Santos J.M., Deshmukh H., Elmassry M.M., Yakhnitsa V., Ji G., Kiritoshi T., Presto P., Antenucci N., Liu X., Neugebauer V. (2024). Beneficial Effects of Ginger Root Extract on Pain Behaviors, Inflammation, and Mitochondrial Function in the Colon and Different Brain Regions of Male and Female Neuropathic Rats: A Gut–Brain Axis Study. Nutrients.

[110] Shen C.L., Wang R., Santos J.M., Elmassry M.M., Stephens E., Kim N., Neugebauer V. (2024). Ginger Alleviates Mechanical Hypersensitivity and Anxio-Depressive Behavior in Rats with Diabetic Neuropathy through Beneficial Actions on Gut Microbiome Composition, Mitochondria, and Neuroimmune Cells of Colon and Spinal Cord. Nutrients.

[111] National Center for Biotechnology Information PubChem Compound Database. https://pubchem.ncbi.nlm.nih.gov/.

[112] Derry S., Rice A.S., Cole P., Tan T., Moore R.A. (2017). Topical Capsaicin (High Concentration) for Chronic Neuropathic Pain in Adults. Cochrane Database Syst. Rev..

[113] Chang A., Rosani A., Quick J. (2024). Capsaicin. StatPearls.

[114] Caillaud M., Aung Myo Y.P., McKiver B.D., Warncke U.O., Thompson D., Mann J., Del Fabbro E., Desmoulière A., Billet F., Damaj M.I. (2020). Key Developments in the Potential of Curcumin for the Treatment of Peripheral Neuropathies. Antioxidants.

[115] Tabanelli R., Brogi S., Calderone V. (2021). Improving Curcumin Bioavailability: Current Strategies and Future Perspectives. Pharmaceutics.

[116] Lee G., Kim S.K. (2016). Therapeutic Effects of Phytochemicals and Medicinal Herbs on Chemotherapy-Induced Peripheral Neuropathy. Molecules.

[117] Brown K., Theofanous D., Britton R.G., Aburido G., Pepper C., Sri Undru S., Howells L. (2024). Resveratrol for the Management of Human Health: How Far Have We Come? A Systematic Review of Resveratrol Clinical Trials to Highlight Gaps and Opportunities. Int. J. Mol. Sci..

[118] Tomé-Carneiro J., Larrosa M., González-Sarrías A., Tomás-Barberán F.A., García-Conesa M.T., Espín J.C. (2013). Resveratrol and Clinical Trials: The Crossroad from In Vitro Studies to Human Evidence. Curr. Pharm. Des..

[119] Amin M., Marouf B.H., Namiq H. (2023). The Effects of Resveratrol Supplementation on the Quality of Life of Diabetic Patients with Neuropathy: Small Randomized Clinical Trial. Iraqi J. Pharm. Sci..

[120] Scaturro D., Vitagliani F., Tomasello S., Sconza C., Respizzi S., Letizia Mauro G. (2023). Combined Rehabilitation with Alpha Lipoic Acid, Acetyl-L-Carnitine, Resveratrol, and Cholecalciferol in Discogenic Sciatica in Young People: A Randomized Clinical Trial. Medicina.

[121] Bagheri Bavandpouri F.S., Azizi A., Abbaszadeh F., Kiani A., Farzaei M.H., Mohammadi-Noori E., Fakhri S., Echeverría J. (2024). Polydatin Attenuated Neuropathic Pain and Motor Dysfunction Following Spinal Cord Injury in Rats by Employing Its Anti-inflammatory and Antioxidant Effects. Front. Pharmacol..

